# Sex hormones, the gut microbiome, and neurodegenerative diseases: Lifespan perspective

**DOI:** 10.4103/NRR.NRR-D-25-00932

**Published:** 2025-12-30

**Authors:** Sarah Stahlke, Carsten Theiss

**Affiliations:** 1 Department of Cytology, Institute of Anatomy, Medical Faculty, Ruhr-University Bochum, Bochum, Germany; 2 International Graduate School of Neuroscience (IGSN), Ruhr-University Bochum, Bochum, Germany

**Keywords:** enteric nervous system, gut–brain axis, lifespan, neurodegenerative diseases, progesterone, steroid hormones

## Abstract

The gut–brain axis represents a highly integrated communication network, connecting the gastrointestinal tract and the central nervous system via neural, immune, endocrine, and metabolic pathways. Steroid hormones, such as estrogens, androgens, and glucocorticoids, play a pivotal role in modulating these interactions across the lifespan. These hormones influence the composition of microbiota, intestinal permeability, and neuroimmune responses, thereby shaping brain function and behavior. Emerging evidence suggests a correlation between disruptions in the gut–brain axis and the onset and progression of neurodegenerative diseases, including Parkinson’s disease, Alzheimer’s disease, and multiple sclerosis. The diseases exhibit distinct sex-specific patterns in terms of prevalence, symptomatology, and progression. These patterns are often the consequence of differences in steroid hormone levels, receptor distribution, and immune responses. Despite these differences, the role of sex as a biological variable remains underrepresented in experimental and clinical research. This review synthesizes current evidence on how steroid hormones modulate gut–brain axis interactions and how these mechanisms contribute to neurodegeneration in a sex-specific manner. We highlight recent findings on hormonal regulation of the gut microbiome and its impact on neuroinflammation and neuronal vulnerability. This overview focuses not only on Parkinson’s disease, in which genetic variations in the gene for brain-derived neurotrophic factor have been observed among others as triggers for dopaminergic neurodegeneration. In addition, Alzheimer’s disease and multiple sclerosis are also considered, in which the prevalence of intestinal dysbiosis and impaired intestinal barrier function have been identified as significant influencing factors. This review provides a comprehensive framework for understanding the gender-specific neurobiology of gut–brain axis by integrating perspectives from the fields of endocrinology, neuroimmunology, and microbiome research. It is argued that a targeted investigation of the interactions between hormones and gut–brain axis is essential for the development of sex-specific therapeutic strategies for neurodegenerative diseases.

## Introduction

The gut–brain axis (GBA) is a complex interface between the endocrine, immune, and nervous systems, enabling bidirectional communication between the gastrointestinal tract and the central nervous system (Cryan et al., 2012; Mayer et al., 2014). It plays a central role in maintaining homeostasis and regulating processes such as digestion, immune response, and behavior (Carabotti et al., 2015). The GBA operates through neural (e.g., the vagus nerve), endocrine (hormones), immune (cytokines), and metabolic pathways, which collectively coordinate physiological function (Foster et al., 2017). Understanding its mechanisms is therefore crucial for elucidating impacts on health and disease (Aburto et al., 2024).

Steroid hormones, including glucocorticoids, estrogens, and androgens, are key modulators of the GBA across the lifespan (Bao et al., 2010; Jaggar et al., 2020). Synthesized in the adrenal glands, gonads, and brain, these hormones act via widely distributed receptors to regulate stress response, metabolism, immune function, and neural activity (McEwen et al., 2015; Chakraborty et al., 2021). They also shape GBA interactions by influencing gut microbiota composition, intestinal permeability, and immune responses, thereby affecting brain function and behavior (Gareau et al., 2008; Clarke et al., 2012).

Sex differences in hormone levels, receptor distribution, and signaling pathways lead to distinct physiological and pathological outcomes (Gillies et al., 2010; Beery et al., 2011; Clayton et al., 2014). For example, males and females differ in susceptibility to neurodegenerative diseases such as Parkinson’s disease (PD), Alzheimer’s disease (AD), and multiple sclerosis (MS). PD involves progressive degeneration of dopaminergic neurons in the substantia nigra, leading to motor and non-motor symptoms (Poewe et al., 2017), and hormonal modulation of the GBA may contribute to the sex-specific prevalence and progression (Bourque et al., 2009; Gillies et al., 2010).

Similarly, AD and MS have been linked to GBA dysfunction. AD is characterized by the accumulation of amyloid-beta plaques and tau tangles, leading to cognitive decline and memory loss (Selkoe et al., 2016). Research has demonstrated that the composition of gut microbiota and intestinal permeability are altered in AD patients, suggesting a potential role of the GBA in disease progression (Harach et al., 2017; Wu et al., 2021). A similar association has been observed in MS, an autoimmune disorder affecting the central nervous system. MS has been linked to changes in gut microbiota and increased intestinal permeability, suggesting a potential role for the GBA in the pathogenesis of this disorder (Berer et al., 2017; Mirza et al., 2017).

Beyond neurodegeneration, sex differences in GBA modulation have recently been summarized in ischemic stroke (Caldarelli et al., 2024), metabolic diseases (Santos-Marcos et al., 2023), and mental health (Leao et al., 2025), emphasizing the importance of sex-sensitive research.

**Figure 1:**
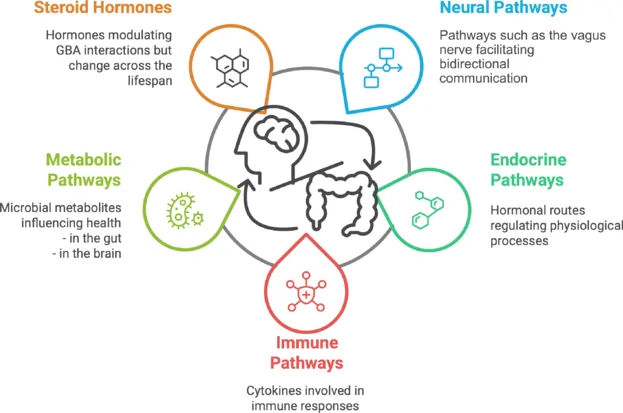
Hormones modulating the gut–brain axis (GBA). Generated with Napkin.AI (https://www.napkin.ai).

This review aims to examine how steroid hormones modulate the GBA and influence brain function in neurodegenerative processes (**Figure 1**). We focus on four key areas: (1) the GBA as a dynamic interface linking the endocrine, immune, and nervous systems; (2) the role of steroid hormones in shaping GBA interactions across the lifespan; (3) sex-specific differences in neurodegenerative diseases, including PD, AD, and MS, as sex differences have also been recently summarized in ischemic stroke (Caldarelli et al., 2024), metabolic diseases (Santos-Marcos et al., 2023), and mental health (Leao et al., 2025); and (4) the implications of hormonal modulation for brain function and potential therapeutic strategies.

## Search Strategy

A comprehensive literature search was conducted in PubMed, Scopus, and Web of Science for publications from January 2002 to August 2025. Search terms included “gut–brain axis,” “sex differences,” “neurodegenerative diseases,” and “steroid hormones.” Only peer-reviewed articles in English focusing on clinical trials and meta-analyses were included, while case reports and non-peer-reviewed studies were excluded. Titles, abstracts, and full texts were screened independently by two reviewers, and any discrepancies were resolved through discussion.

## Steroid Hormones and the Gut–Brain Axis

Steroid hormones, including progesterone and testosterone, play a crucial role in modulation of the gut microbiome and immune system. Evidence from both animal and human studies suggests that these hormones influence microbial diversity, intestinal permeability, and systemic immune responses. Importantly, much of the mechanistic insight derives from animal models, while human data remains more correlative and limited in scope. Progesterone has been shown in murine models to promote the growth of beneficial bacteria, such as Lactobacillus species (Sovijit et al., 2021), while concomitantly reducing gut permeability for translocation of harmful bacteria (Zhou et al., 2019). In humans, pregnancy provides indirect evidence of progesterone-mediated microbial shifts, though causal pathways remain incompletely defined (Sinha et al., 2023). Testosterone, has been linked in animal models to enhanced microbial diversity and the production of short-chain fatty acids (SCFAs), which support gut health and exhibit anti-inflammatory properties (Canfora et al., 2015; Campos-Perez et al., 2021). Recent human data also indicate associations between circulating testosterone levels and microbiome composition; in a study of orchiectomized mice supplemented with testosterone, microbial diversity decreased and Firmicutes/Bacteroidetes ratios shifted, and parallel observational findings in men suggest comparable trends (Song et al., 2023). These associations, however, do not establish causality.

Neufeld et al. (2024) describe the impact of dietary factors and microbiota composition on PD, emphasizing the potential of microbiota-based interventions, such as the Mediterranean diet, to mitigate disease risk and provide novel therapeutic insights.

SCFAs, including butyrate, propionate, and acetate, function as energy sources for colonocytes and regulate local and systemic immune responses. The interaction between steroid hormones and SCFAs further illustrates the bidirectional nature of the GBA. SCFAs regulate hormone receptor expression and can modulate synthesis of glucocorticoids, estrogens, and androgens (Canfora et al., 2015). For example, butyrate enhances glucocorticoid receptor expression in the gut (Fukumoto et al., 2003). While these mechanisms are robustly demonstrated in preclinical models, clinical validation remains sparse.

Neuroprotective effects of hormones have also been proposed. As demonstrated by Stegemann et al. (2023), in rats, progesterone has been shown to exert neuroprotective effects on the enteric nervous system in the context of PD. Using quantitative reverse transcription polymerase chain reaction and immunofluorescence, they identified various progesterone receptors (PR-A/B, mPRa, mPRb, and PGRMC1) in rat enteric nervous system neurons and found that progesterone treatment reduced rotenone-induced neuronal cell death by 45%, with the PGRMC1 receptor playing a crucial role. These findings suggest that progesterone-based therapies could serve as potential strategies for neurodegenerative diseases originating in the enteric nervous system. Although compelling, these findings await translational confirmation in human studies.

The influence of hormones on gut permeability and inflammation emphasizes the importance of endocrine-microbiome interactions. Estrogens, for instance, have been shown to enhance gut barrier integrity by upregulating tight junction proteins, thereby preventing harmful bacterial translocation and reducing systemic inflammation (Braniste et al., 2014; Chelakkot et al., 2018). Conversely, glucocorticoids can impair gut barrier function, increase permeability and promote inflammation, a mechanism implicated in various gastrointestinal and neurodegenerative disorders (Fasano, 2011; Tena-Garitaonaindia et al., 2022). Human stress studies confirm correlations between elevated glucocorticoids and increased intestinal permeability, although direct causal links to neurodegeneration are not yet established. Additionally, progesterone and testosterone contribute to immune modulation within the gut. Progesterone inhibits pro-inflammatory cytokines such as tumor necrosis factor alpha (TNF-α) and interleukin (IL)-6 (Coronel et al., 2016; Zhou et al., 2019) while promoting anti-inflammatory cytokines such as IL-10 (Bommer et al., 2016). Testosterone exerts a similar influence on immune responses by reducing T-helper 1 (Th1) cell activation and enhancing regulatory T cell (Treg) activity, thereby contributing to the maintenance of immune tolerance and preventing excessive inflammation (Ben-Batalla et al., 2020; Jones et al., 2020). These hormonal interactions underscore their critical role in maintaining gut homeostasis and influencing the pathophysiology of neurodegenerative diseases. Song et al. (2023) examined the relationship between testosterone and gut microbiome composition, with particular attention to testosterone receptor expression in the intestine. They found that changes in testosterone levels were associated with alterations in gut microbiome composition. In orchiectomized mice, microbial diversity increased, while testosterone supplementation was linked to decreased diversity and changes in the *Firmicutes*/*Bacteroidetes* ratio. Testosterone administration was furthermore found to be associated with an increased presence of certain opportunistic bacteria. These mechanisms are well supported in experimental models, but larger-scale human cohort studies are still needed to confirm their clinical relevance.

Another study addresses the presence and potential influence of estrogen receptors (ER) in intestinal tissue, focusing specifically on their impact on hormone production and the intestinal microbiome (Kluxen et al., 2012). The study found that both ER-alpha and ER-beta are expressed in the small intestine and colon of female rats. Activation of these receptors showed different effects on cell proliferation and apoptosis. Specifically, ER-alpha activation increased both proliferation and apoptosis in the small intestine and colon, while ER-beta activation decreased proliferation and increased apoptosis in these regions. Moreover, cadmium exposure was found to inhibit ER-beta mRNA and protein expression in intestinal tissue. It should be noted, however, that these findings are based solely on female rats. Therefore, potential sex-specific differences in ER-alpha and ER-beta expression or function cannot be addressed in the present study, and future work will be required to determine whether similar patterns are observed in males.

The study did not directly examine the effects of intestinal ER expression on hormone production or the microbiome, but it suggested potential implications for local hormone signaling within the intestine. Cadmium was shown to modulate ER expression and signaling, inducing C3-mRNA and PR-protein expression in a dose-dependent manner. An ER-antagonist prevented cadmium-induced expression, indicating an ER-mediated mechanism. These findings imply that changes in ER expression or activation in the intestine could influence local hormone-responsive gene expression. However, the available evidence is limited to a single study, which restricts the ability to draw broader conclusions about the effects of estrogen on the gut. Further research is needed to determine whether intestinal ER expression influences hormone production or the intestinal microbiome.

Collectively, these findings illustrate the complex interplay between steroid hormones, gut microbiota, and the immune system in the context of the GBA, as summarized in **Table 1**. Understanding these mechanisms may allow for the development of novel therapeutic strategies for neurodegenerative and inflammatory disorders.

**Table 1:** Modulators of the gut–brain axis

Modulator	Effect	Mechanism	Reference
SCFA			
Butyrate	↑ Glucocorticoid receptor expression in the gut	Epigenetic modulation of receptor expression	Fukumoto et al., 2003
Steroid hormones			
Estrogens	↑ Gut barrier integrity ↓ Bacterial translocation ↓ Inflammation	Upregulation of tight junction proteins	Braniste et al., 2014; Chelakkot et al., 2018
	ER-α: ↑ Proliferation ↑ Apoptosis ER-β: ↓ Proliferation ↑ Apoptosis	Differential activation of ER subtypes in intestinal cells	Kluxen et al., 2013
Glucocorticoids	↑ Gut permeability ↑ Inflammation ↑ Neurodegeneration	Downregulation of tight junction proteins, immune modulation	Fasano, 2011; Tena-Garitaonaindia et al., 2022
Progesterone	↑ Growth of Lactobacillus ↓ Translocation of harmful bacteria	Reducing gut permeability	Zhou et al., 2019
	↓ Pro-inflammatory cytokines (tumor necrosis factor-α, interleukin-6) ↑ Anti-inflammatory cytokines (interleukin-10)	Suppression of inflammatory pathways	Bommer et al., 2016; Coronel et al., 2016
	↑ Neuroprotection in enteric nervous system ↓ Rotenone-induced neuronal death	Activation of PGRMC1 receptor	Stegemann et al., 2023
Testosterone	↑ Microbial diversity ↑ SCFA production	Alteration of gut microbiota metabolism	Canfora et al., 2015; Campos-Perez et al., 2021
	↓T-helper 1 activation ↑Regulatory T cell activity ↑ Immune tolerance	Modulation of T cell responses	Ben-Batalla et al., 2020; Jones et al., 2020
	Alters gut microbiome composition, affects Firmicutes/Bacteroidetes ratio	Regulation of gut microbial communities	Song et al., 2023

ER: Estrogen receptor; PGRMC1: progesterone receptor membrane component 1; SCFA: short-chain fatty acids.

### Sex differences in gut–brain communication

Sex-specific differences in gut–brain communication are evident in various aspects of GBA interactions, including vagal signaling and gut-derived neurotransmitters (**Table 2**). The vagus nerve, a critical component of the GBA, exhibits sex-specific differences in its structure and function. For instance, preclinical studies demonstrate that female rats have a higher density of vagal afferent fibers in the gut compared to males, which may contribute to differences in gut–brain signaling (Asarian et al., 2013). Human studies likewise suggest sex differences in vagal tone and stress responses, though mechanistic pathways remain incompletely understood (Breit et al., 2018; Holingue et al., 2020).

**Table 2:** Sex-biased mechanisms of gut–brain communication and disease outcomes

Mechanism	General (both sexes)	Predominantly female-biased effects	Predominantly male-biased effects	Implications for disease outcomes
Neurotrans-mitter modulation by steroid hormones	Sex hormones modulate release of gut-derived neurotransmitters including serotonin and GABA, influencing mood and behavior (Barth et al., 2015)	Estrogens ↑ serotonin synthesis/release in gut → impact on central serotonergic activity, mood and anxiety (Bethea et al., 2002)	Testosterone modulates GABAergic signaling in the gut → effects on stress resilience and emotional regulation (Frye et al., 2008)	Sex differences in stress responses, mood regulation, and vulnerability to neurodegeneration
Vagal innervation	Vagal afferents mediate gut–CNS signaling; density differs between sexes (Gillies et al., 2010)	Higher vagal afferent density reported in females → enhanced gut–brain signaling (Gillies et al., 2010)		May contribute to female-biased prevalence of anxiety and altered gut–brain signaling
Gut microbiota interactions	Hormones modulate microbiota composition across lifespan (Mallott et al., 2020)	Pregnancy-related hormonal shifts induce microbiota changes and enhance gut barrier to protect fetus (Mallott et al., 2020)	Testosterone influences microbiota diversity and immune modulation, possibly linked to physical performance (Geary, 2010)	Microbiota shifts contribute to sex differences in inflammation and neurodegeneration
Evolutionary adaptations	Sex differences in GBA reflect divergent reproductive/survival strategies (Geary, 2010)	Reproductive adaptations: pregnancy/lactation require enhanced gut–brain signaling and immune control (Mallott et al., 2020)	Male adaptations favor energy balance and stress coping capacities (Geary, 2010)	Distinct trajectories of brain aging and disease vulnerability across sexes
Neurode-generative outcomes	Shared mechanisms: neuroinflammation, oxidative stress, mitochondrial dysfunction (Gillies et al., 2010)	↑ Prevalence of anxiety/depression in women; postmenopausal estrogen decline linked to ↑ AD risk (Gillies et al., 2010)	↑ Susceptibility of men to PD and certain other neurodegenerative disorders (Gillies et al., 2010)	Sex-biased incidence and progression of AD, PD, and MS

AD: Alzheimer’s disease; CNS: central nervous system; GABA: gamma-aminobutyric acid; GBA: gut–brain axis; MS: multiple sclerosis; PD: Parkinson’s disease.

Sex hormones can also modulate the release of gut-derived neurotransmitters, including serotonin and gamma-aminobutyric acid (GABA), which are known to play essential roles in regulating mood and behavior (Barth et al., 2015). Estrogens have been shown to increase the synthesis and release of serotonin in the gut, which can influence central serotoninergic activity and contribute to variations in mood and anxiety levels (Bethea et al., 2002). Testosterone, on the other hand, has been found to modulate GABAergic signaling in the gut, which can impact stress resilience and emotional regulation (Frye et al., 2008). While these mechanisms are well established in animal models, human data are mostly correlative, underscoring the need for clinical studies.

The gut microbiota can influence hormone levels, while hormones can shape microbial composition (Neuman et al., 2015; He et al., 2021). Certain bacteria possess genes capable of producing estrogen-metabolizing enzymes, allowing them to modulate serum estrogen levels (Fernández et al., 2018). Conversely, estrogen-like compounds may promote the growth of specific bacterial species (Fernández et al., 2018). It is generally accepted that microbiota have the capacity for the metabolic transformation of steroid hormones and subsequent degradation (Hussain et al., 2021). One example is *Akkermansia muciniphila*, an estrogen-responsive microbiota that is decreased in gonadectomized female mice (Sakamuri et al., 2023).

From an evolutionary perspective, male and female GBA interactions may exhibit divergent patterns, consequent to distinct reproductive and survival strategies. To illustrate, females might have undergone an evolutionary process that led to the development of more robust gut-brain communication mechanisms to support pregnancy and lactation. Such processes require precise regulation of energy balance, as well as immune function. During pregnancy, hormonal changes can alter gut microbiota composition and enhance gut barrier function, probably to protect the developing fetus from harmful pathogens (Mallott et al., 2020). In contrast, males may have developed different GBA interactions to optimize physical performance and stress responses, which are crucial for survival and reproduction (Geary, 2010). The effects of testosterone on gut microbiota diversity and immune modulation may have evolved to enhance the ability of males to cope with physical stress and infections. These evolutionary perspectives underscore the importance of considering sex differences in GBA research and their implications for health and disease. Understanding how sex-specific hormonal modulation of the GBA influences brain function and behavior can provide valuable insights into the mechanisms underlying neuropsychiatric and neurodegenerative disorders. For instance, the heightened prevalence of anxiety and depression in women and the increased vulnerability of men to certain neurodegenerative diseases, such as PD, may be attributable to sex differences in gut–brain communication (Gillies et al., 2010). However, the majority of current data remains associative, and longitudinal human studies will be essential to clarify causal links.

## Hormonal Changes Across the Lifespan and Their Impact on the Gut–Brain Axis

**Figure 2:**
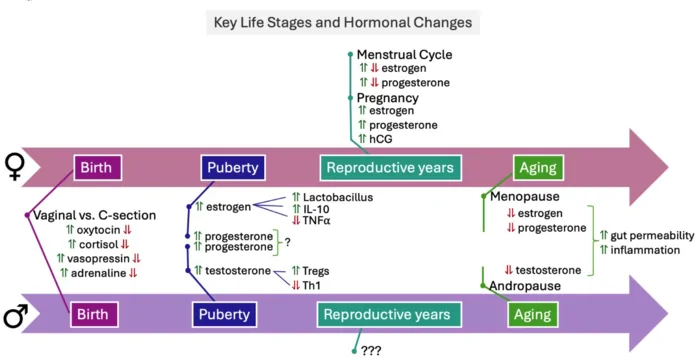
Hormonal changes during key life stages. hCG: Human chorionic gonadotropin; IL: interleukin; Th1: T-helper 1; TNF: tumor necrosis factor; Tregs: regulatory T cells.

Hormones fluctuate across the lifespan, shaping the GBA through interactions with the microbiome, immune system, and neural circuits. Key transitions occur at birth, during puberty and early adulthood, throughout the reproductive years (including pregnancy), and with aging (**Figure 2**). While animal studies provide mechanistic insight, most human data remain associative, and large-scale longitudinal validation is still needed.

### Birth, puberty, and early adulthood

Hormonal fluctuations during childbirth differ between vaginal delivery and cesarean (c-) section, potentially influencing the gut–brain axis throughout life (Sandall et al., 2018). Vaginal delivery triggers significant surges in oxytocin and cortisol, which facilitate neonatal adaptation and may promote beneficial gut microbiota colonization, thereby supporting optimal gut–brain communication. In contrast, c-sections are associated with altered hormonal responses and distinct microbial exposures, which might adversely affect the development of the gut-brain axis (Palmer et al., 2007; Biasucci et al., 2010; Dominguez-Bello et al., 2010). These early-life hormonal and microbial variations could have enduring effects on neurodevelopment and stress-related disorders.

During puberty and early adulthood, significant hormonal changes drive shifts in gut microbiota composition and neuroimmune balance. The surge in sex hormones, such as estrogen and testosterone, leads to the maturation of the reproductive system and secondary sexual characteristics (Alsaker et al., 2020). These hormonal changes also influence the gut microbiota, promoting the growth of specific bacterial species and altering the overall microbial diversity (Calcaterra et al., 2022). Human studies report that estrogen increases the abundance of bacteria producing short-chain fatty acids, which play a crucial role in maintaining gut health and modulating immune responses (Sun et al., 2018).

Experimental evidence indicates that the hormonal shifts during puberty also impact the neuroimmune balance by modulating the activity of immune cells and the production of cytokines (Morales-Montor et al., 2011). Estrogen, for example, has anti-inflammatory properties, enhancing the production of anti-inflammatory cytokines such as IL-10, while reducing pro-inflammatory cytokines, including TNF-α (Shivers et al., 2015). Testosterone, on the other hand, suppresses the activity of Th1 cells and promotes the differentiation of regulatory T cells (maintaining immune tolerance and preventing excessive inflammation (Kissick et al., 2014). These findings are mechanistically plausible, but direct causal confirmation in humans remains limited.

Sex differences in stress responses and neurodevelopmental outcomes further emerge during adolescence. Females generally exhibit higher levels of stress-induced glucocorticoid release, which can influence gut-brain signaling and neurodevelopment (Holingue et al., 2020). Chronic stress during this critical period can lead to alterations in gut microbiota composition, increased gut permeability, and systemic inflammation, potentially contributing to neuropsychiatric disorders such asanxiety and depression (Foster et al., 2017; Esposito et al., 2022). Additionally, the maturation of brain regions involved in emotional regulation, such as the prefrontal cortex and amygdala, exhibits sex-specific differences, resulting in distinct neurodevelopmental outcomes for males and females (Frere et al., 2020). These associations highlight plausible pathways, but causality requires confirmation through longitudinal human studies.

### Reproductive years and pregnancy

The reproductive years, including pregnancy, are marked by fluctuating hormone levels that significantly impact gut-brain signaling. During the menstrual cycle, variations in estrogen and progesterone influence gut motility, microbiota composition, and immune function, with high progesterone levels in the luteal phase associated with slower gut transit in humans (Yang et al., 2006). Pregnancy induces profound hormonal changes, including elevated levels of estrogen, progesterone, and human chorionic gonadotropin, which modulate gut microbiota composition and the gut-brain axis (Nuriel-Ohayon et al., 2019). These hormonal shifts promote the growth of specific bacterial species that enhance nutrient absorption and energy storage, thereby supporting fetal development (Sun et al., 2018; Feng et al., 2025). Preclinical studies suggest that pregnancy-associated changes in gut microbiota may have neuroprotective potential, as certain microbial metabolites, such as SCFAs, can cross the blood–brain barrier and exert anti-inflammatory and neuroprotective effects (Dong et al., 2024; Liu et al., 2025). Fluctuating hormone levels during pregnancy also impact the maternal immune system, promoting a state of immune tolerance to protect the developing fetus (Krupa et al., 2024). Progesterone, for example, has immunomodulatory effects that reduce the activity of pro-inflammatory immune cells and enhance the production of anti-inflammatory cytokines (Lei et al., 2014; Coronel et al., 2016; Krupa et al., 2024). These immune changes can influence gut-brain signaling and may contribute to the reduced risk of certain neuroinflammatory or neuropsychiatric disorders during pregnancy, such as MS (Voskuhl et al., 2022).

Epidemiological studies suggest that reproductive history influences long-term risk of neurodegenerative disease. Key findings include that women with longer fertile lives (e.g., late menopause) and higher parity (more pregnancies) appear to have a delayed onset of PD. Additionally, there is a notable sex prevalence for AD, with women being at a higher risk. This suggests that hormonal changes during pregnancy and menopause could impact disease susceptibility and progression (Lv et al., 2017). Longer cumulative lengths of pregnancies and extended exposure to endogenous estrogens have been associated with a delayed onset of PD, suggesting a protective role of estrogens (Ragonese et al., 2004; Yadav et al., 2012). This may extend to other neurodegenerative diseases, although specific studies are limited. Gestational factors, including maternal diet and stress, can influence brain development and potentially predispose individuals to neurodegenerative diseases, such as AD, later in life (Natale et al., 2023). Overall, hormonal factors related to fertility, particularly estrogen exposure, appear to play a significant role in influencing the risk and progression of neurodegenerative diseases beyond PD. The protective effects of estrogens and the impact of reproductive history highlight the importance of considering sex-specific factors in understanding and potentially mitigating the risk of these diseases. While suggestive of causality, most findings remain associative and call for validation in prospective human cohorts.

### Aging, menopause, and andropause

Aging is associated with a decline in sex hormones, such as estrogen and testosterone, which can result in increased gut permeability and inflammation. Menopause, characterized by a substantial decrease in estrogen levels, is associated with alterations in gut microbiota composition and increased intestinal permeability (Peters et al., 2022). Observational studies suggest that these changes may facilitate the translocation of microbial products into circulation, potentially promoting systemic inflammation and contributing to cognitive decline or neurodegenerative processes, such as AD (Rizzetto et al., 2018). This increased inflammation can have detrimental effects on brain function and may contribute to the development of neurodegenerative diseases, such as AD (Braniste et al., 2014). While animal studies support causative mechanisms linking estrogen loss to gut and brain dysfunction, direct causal evidence in humans is limited.

In men, andropause, sometimes referred to male equivalent of menopause, is characterized by a gradual decline in testosterone levels, which can also impact both gut health and inflammation. Reduced testosterone levels have been associated with changes in gut microbiota composition, increased gut permeability, and elevated levels of pro-inflammatory cytokines (Mohamad et al., 2018). These changes have the potential to affect brain function and may contribute to the increased risk of neurodegenerative diseases, such as PD, in aging men (Gillies et al., 2010).

### Hormonal replacement therapy

Hormone replacement therapy (HRT) has been explored as a potential intervention to mitigate the effects of declining sex hormones on the GBA and brain function. Estrogen replacement therapy in postmenopausal women has been shown to restore gut barrier function, reduce inflammation, and improve cognitive function in postmenopausal women (Brinton, 2009). In a similar fashion, testosterone replacement therapy in aging men has been associated with improvements in gut microbiota composition, reduced gut permeability, and enhanced cognitive performance (Jung et al., 2016). However, the long-term effects and safety of HRT on the GBA and brain function require further investigation. Studies examining the relationship between menopause, HRT, and neurodegenerative disorders reveal complex interactions influenced by hormone types, duration, and timing of therapy. Some studies associate menopausal hormone therapy with an increased risk of PD, particularly with long-term use of estrogen-progesterone combinations or tibolone (Yuk et al., 2023). Estrogen-only therapies were linked to a higher PD risk in women with hysterectomy, especially with extended use (Popat et al., 2005). Conversely, experimental models have demonstrated potential neuroprotective effects of estrogen, possibly by mitigating dopaminergic neuron loss and reducing neuroinflammation (Labandeira-Garcia et al., 2016). Women with prolonged estrogen exposure, such as late menopause, showed reduced PD risk (Lv et al., 2017). An epidemiological study reports inconsistent findings on the role of estrogen replacement therapy. While some suggested reduced PD risk with estrogen replacement therapy, others found no clear benefits (Ragonese et al., 2004). Surgical menopause (e.g., bilateral oophorectomy) is associated with higher PD risk compared to natural menopause. This risk may be mitigated by HT initiated shortly after surgery (Ibrahim et al., 2022). The “critical window hypothesis” suggests that starting HT shortly after menopause may provide neuroprotective benefits, while delayed initiation could potentially be ineffective or even harmful (Rodriguez-Perez et al., 2010).

The relationship between HRT and neurodegenerative diseases extends beyond PD and includes both AD and dementia. Some studies indicate a significant association between hormone therapy and an increased risk of AD and all-cause dementia, particularly with combined estrogen-progestogen formulations (Popat et al., 2005; Wu et al., 2020). Conversely, other research suggests that estrogen replacement therapy may decrease the risk of AD, with some meta-analyses showing a reduced risk of onset and development. The neuroprotective effects of estrogens, particularly 17β-estradiol, have been observed in several studies, although the results remain inconclusive (Brinton, 2009; Scott et al., 2012). The impact of HRT on AD risk may depend on the timing of initiation, type of hormone used, and individual characteristics, including genetic background and cardiovascular health (Lv et al., 2017; Wu et al., 2020).

In summary, while hormone therapy, particularly estrogen-based, shows potential for neuroprotection, its influence on neurodegenerative disease risk is influenced by factors such as timing, type of therapy, and individual hormonal history. The impact of HRT is determined by numerous factors, including age, duration of therapy, and individual health characteristics, making it challenging to draw definitive conclusions. Further longitudinal studies and well-controlled clinical trials are required to clarify how hormone replacement might be optimized to support GBA function and reduce neurodegenerative disease risk.

## Sex Bias in Basic and Clinical Research

Sex bias in basic and clinical research has been a longstanding problem with significant consequences for our understanding of health and disease, including disorders involving the GBA. Historically, biomedical research has predominantly focused on male subjects, both in animal studies and clinical trials, leading to a significant gap in knowledge regarding female physiology and the influence of sex hormones on various biological processes (Beery et al., 2011; Clayton et al., 2014). This bias is particularly relevant for studies of the GBA and its role in neurodegenerative diseases, as sex differences in hormonal regulation, immune responses, and microbiota composition are critical factors that influence GBA interactions.

One of the main reasons for sex bias in research is the perceived complexity and variability introduced by the female reproductive cycle. Researchers have often excluded female subjects to avoid the fluctuations in hormone levels associated with the menstrual cycle, which can affect experimental outcomes (Beery et al., 2011). However, this approach overlooks the fact that these hormonal fluctuations are a fundamental aspect of female biology and can provide valuable insights into how sex hormones modulate physiological processes, including those related to the GBA. The exclusion of female subjects from research has led to a lack of understanding of how sex hormones influence gut microbiota composition and gut-brain signaling. For instance, studies have shown that estrogen and progesterone can significantly alter the gut microbiota, promote the growth of beneficial bacteria, and modulate immune responses (Zhou et al., 2019). These hormonal effects are crucial for understanding sex-specific differences in the GBA and their implications for health and disease. Without adequate representation of female subjects, our knowledge of these interactions remains incomplete, leading to at best suboptimal and at worst potentially harmful treatment strategies for women.

In clinical research, the underrepresentation of women in clinical trials has resulted in a lack of sex-specific data on the safety and efficacy of medical treatments. This bias can have serious consequences, as men and women may respond differently to medications due to differences in hormone levels, body composition, and metabolism (Clayton et al., 2014). For example, women are more likely to experience adverse drug reactions and may require different dosages or treatment regimens compared to men (Madla et al., 2021). Addressing this bias is essential for the development of personalized medicine approaches that take into account sex-specific differences in disease presentation and treatment response. However, most of these mechanistic insights are derived from animal studies, and translation to humans remains incomplete. Large-scale cohort studies in women are still scarce, and it is important to emphasize that current evidence in humans remains largely associative rather than causal.

The sex bias in research also extends to the study of neurodegenerative diseases, such as PD, AD, and MS. These diseases exhibit sex-specific differences in prevalence, progression, and response to treatment, which are influenced by hormonal regulation of the GBA (Gillies et al., 2010; Holingue et al., 2020; Jaggar et al., 2020). For instance, women are more likely to develop AD, while men have a higher risk of developing PD (Wooten, 2004; Gillies et al., 2010). Understanding the role of sex hormones in modulating GBA interactions is crucial for elucidating the mechanisms underlying these differences and developing effective therapeutic strategies. To address this sex bias in research, several initiatives have been implemented to promote the inclusion of female subjects in both basic and clinical studies. This shift is essential for advancing mechanistic understanding while ensuring clinical-translational relevance. Incorporating gender-sensitive perspectives into GBA research will enable more robust identification of causal pathways, support the development of personalized therapies, and ultimately reduce the risk of misinformed or suboptimal treatments for women.

### Incorporating sex-specific mechanisms into neurodegenerative disease models

**Figure 3:**
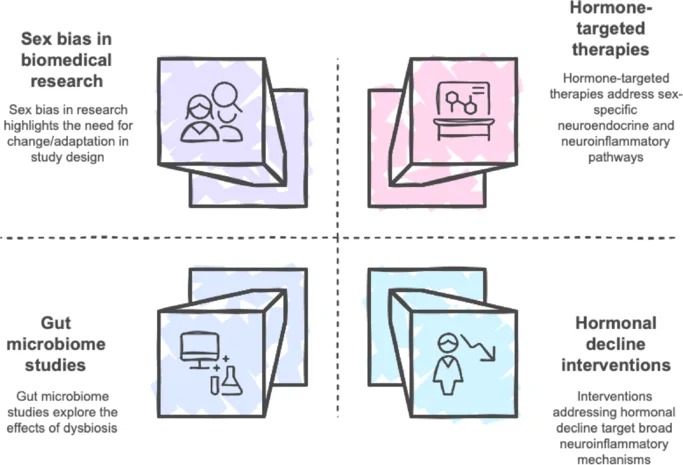
Strategic approaches to integrating sex as a biological variable in neurodegenerative disease research. Created with Napkin.AI.

Adapting neurodegenerative disease models to account for sex-specific mechanisms is crucial for understanding the different progression and response to treatment in men and women (**Figure 3**). Preclinical animal models should include both sexes and consider the impact of sex hormones on disease pathology. For example, incorporating ovariectomized and orchiectomized animal models can help study the effects of estrogen and testosterone depletion, respectively, on neurodegenerative processes (Gillies et al., 2010). Additionally, developing sex-specific cell culture models, such as induced pluripotent stem cells derived from male and female patients, offers an opportunity to reduce reliance on animal models and investigate molecular mechanisms in a human-specific context (Kiris, 2022).

## Neurodegenerative Processes and the Gut–Brain–Hormone Interaction

The interplay between the gut, brain, and hormones is a critical factor in the development and progression of neurodegenerative diseases. This multidirectional interaction is shaped by gut microbiota composition, hormone levels, and immune responses, collectively contributing to neuroinflammation and neuronal damage. While animal models have provided much of the mechanistic framework, human data, especially from prospective cohorts and clinical studies, remain essential to validate these mechanisms and to disentangle causal effects from associations.

### Gut dysbiosis, hormonal decline, and neuroinflammation

Gut dysbiosis, characterized by an imbalance in the composition and function of gut microbiota, has been implicated in the pathogenesis of various neurodegenerative diseases, including PD, AD, and MS (Cryan et al., 2012). Dysbiosis can lead to increased intestinal permeability, also known as “leaky gut,” allowing harmful bacteria and their metabolites to translocate into the bloodstream and trigger systemic inflammation (Fasano, 2011). This systemic inflammation can cross the blood-brain barrier and induce neuroinflammation, a key driver of neurodegenerative processes (Braniste et al., 2014). Another critical aspect of the interplay between gut microbiota and brain function is the influence of microbiota on neuroplasticity, which is a huge field on its own and has been recently reviewed by Al Noman et al. (2025).

Hormonal decline, particularly the reduction of sex hormones such as estrogen and testosterone, further exacerbates both gut dysbiosis and neuroinflammation. While adequate levels of estrogen have been shown to improve gut barrier function by upregulating tight junction proteins, thereby reducing gut permeability and preventing the translocation of pro-inflammatory molecules (Zhou et al., 2017). The decline in estrogen levels during menopause can compromise gut barrier integrity, leading to increased gut permeability and systemic inflammation (Peters et al., 2022). Similarly, the gradual decline in testosterone levels during andropause can alter gut microbiota composition and increase gut permeability, contributing to systemic and neuroinflammation.

The combined effects of gut dysbiosis and hormonal decline create a pro-inflammatory environment that promotes neuroinflammation and neuronal damage. For instance, lipopolysaccharides, endotoxins produced by gram-negative bacteria in the gut, can activate microglia, the resident immune cells in the brain, leading to the release of pro-inflammatory cytokines such as TNF-α and IL-1β (Kalyan et al., 2022). Chronic activation of microglia and sustained neuroinflammation are hallmarks of neurodegenerative diseases and contribute to the progressive loss of neuronal function (Lull et al., 2010). While these pathways are well characterized in animal studies, clinical-translational research is necessary to clarify their relevance in humans and to distinguish associative signals from causal mechanisms.

### Sex-specific pathways in neurodegeneration

Sex-specific pathways play a crucial role in both the onset and progression of neurodegenerative diseases, with distinct differences observed between men and women. These differences are influenced by variations in hormone levels, immune responses, and genetic factors. Importantly, much of the mechanistic understanding comes from preclinical models, while clinical and epidemiological studies increasingly provide evidence of sex-specific trajectories. A careful distinction must therefore be made between associations observed in population-based studies and causal mechanisms inferred from experimental work.

In PD, men are more likely to develop the disease and exhibit faster disease progression compared to women (Gillies et al., 2010). Testosterone has been implicated in the sex-specific differences observed in PD. A preclinical study suggests that testosterone can exert neuroprotective or neurotoxic effects dependent on oxidative stress levels, contributing to the degeneration of dopaminergic neurons in the substantia nigra at high oxidative stress levels (Holmes et al., 2013). In parallel, clinical imaging studies show that men tend to accumulate more iron in the substantia nigra, which can catalyze the production of reactive oxygen species and further promote neuronal damage (Bartzokis et al., 2007). Conversely, epidemiological evidence supports the protective role of estrogen, consistent with experimental findings that estrogen reduces oxidative stress, inhibits apoptosis, and modulates neuroinflammatory responses (Morale et al., 2006). Furthermore, in preclinical studies, estrogen can enhance the expression of neurotrophic factors, such as brain-derived neurotrophic factor, which support neuronal survival and function (Harte-Hargrove et al., 2013). However, while these associations are robust, causality in humans remains to be firmly established.

In AD, women are more likely to develop the disease, particularly after menopause, when estrogen levels decline. Large-scale cohort studies confirm that the loss of estrogen’s protective effects on the brain, combined with age-related changes in gut microbiota composition, may contribute to the increased risk of AD in postmenopausal women (Wu et al., 2020). In an animal study, estrogen has been shown to modulate amyloid-beta metabolism, reduce tau phosphorylation, and enhance synaptic plasticity, all of which are critical factors in AD pathogenesis (Selkoe et al., 2016). The decline in estrogen levels during menopause can disrupt these protective mechanisms, leading to the accumulation of amyloid-beta plaques and tau tangles, which are hallmarks of AD (Selkoe et al., 2016). The loss of protective effects of estrogen may therefore contribute to amyloid and tau pathology, but the causal link in humans remains debated.

In MS, sex differences in disease prevalence and progression are also evident, with women being more likely to develop the disease and men experiencing more severe disease progression (Golden et al., 2017). The hormonal regulation of immune responses plays a significant role in these sex-specific differences. Estrogen has been shown to modulate the activity of immune cells, reducing the production of pro-inflammatory cytokines and promoting the differentiation of regulatory T cells, which help maintain immune tolerance (Alanazi et al., 2024). The decline in estrogen levels during menopause may lead to a dysregulated immune response, contributing to the increased risk of MS relapses in postmenopausal women (Christianson et al., 2015). Nevertheless, the extent to which these immune changes are causal or correlative remains under investigation.

In summary, sex-specific pathways, influenced by variations in hormone levels and immune responses, play a crucial role in the distinct differences observed between men and women in neurodegenerative diseases. Understanding these interactions is essential for developing targeted therapeutic strategies that address the unique physiological and hormonal differences between sexes. The gut–brain–hormone interaction, including the role of gut dysbiosis and hormonal decline, contributes to neuroinflammation and drives neuronal damage and disease progression. Further research is needed to elucidate the complex interplay between these factors and their impact on neurodegenerative diseases.

### Regulation of oxidative stress, mitochondrial function, and inflammatory pathways

Mitochondrial dynamics and responses to oxidative stress are directly influenced by sex hormones. Testosterone and estrogen regulate mitochondrial biogenesis, metabolic activity, and organelle communication, ensuring efficient energy production and cellular homeostasis (Lynch et al., 2021). Furthermore, mitochondria serve as the primary site for steroidogenesis, with the first step of sex hormone synthesis occurring within these organelles, linking mitochondrial function to endocrine regulation (Miller, 2013). Experimental models suggest that estrogens may exert both protective and pro-oxidative effects depending on cellular context: they can activate mitochondrial activity and increase reactive oxygen species, but also enhance antioxidant defense mechanisms (Holmes et al., 2013; Sumien et al., 2021). By modulating cytokine expression and immune signaling pathways, sex hormones regulate inflammation at the molecular level. Testosterone has been shown in preclinical studies to reduce the expression of pro-inflammatory mediators, such as TNF-α in macrophages, thereby exerting an anti-inflammatory effect (Corcoran et al., 2010). In contrast, the influence of estrogen on inflammation appears to be context-dependent, with its impact on C-reactive protein expression in macrophages varying according to extracellular lipid composition (Gilliver, 2010). Additionally, androgens regulate local hormone conversion in synovial cells by inhibiting aromatase activity, thereby preventing excessive estrogen synthesis in inflammatory environments (Capellino et al., 2014).

### Sex hormones, protein homeostasis, and neurodegeneration

Sex hormones modulate pathways of protein aggregation and degradation, which are central to neurodegenerative processes such as PD, AD, and MS (Bourque et al., 2009; Gillies et al., 2010). These effects are mediated in part via the GBA, as hormonal fluctuations influence gut microbiota composition and microbial metabolites, including SCFAs. They can cross the blood–brain barrier and regulate proteostatic machinery, such as the proteasome and autophagy pathways, thereby promoting clearance of misfolded proteins like α-synuclein and tau (Cryan et al., 2012; Westfall et al., 2017).

Estrogens enhance autophagic clearance of misfolded proteins, potentially reducing α-synuclein aggregation in dopaminergic neurons, while testosterone and progesterone may act indirectly via microbiota-mediated immune signaling (Brinton et al., 2009; Jaggaret al., 2020). Dysregulation of protein homeostasis contributes to neurodegeneration, and GBA-mediated hormonal modulation provides a mechanism for sex-specific differences in disease susceptibility and progression. Hormonal fluctuations during the menstrual cycle, pregnancy, menopause, and andropause should therefore be considered in experimental design and interpretation (Clayton et al., 2014).

Advanced techniques such as single-cell RNA sequencing and metabolomics allow detailed mapping of the molecular and cellular mechanisms underlying sex-specific differences in the GBA and proteostasis (McEwen et al., 2015; Lee et al., 2022). Integrating these pathways provides a framework for understanding how steroid hormones interact with gut microbiota and influence neuronal protein homeostasis, thereby affecting neurodegenerative processes across the lifespan (Cryan et al., 2012; Westfall et al., 2017).

### Potential for microbiome-based and hormone-targeted interventions in neurodegeneration

Emerging evidence highlights the therapeutic potential of microbiome modulation and hormone-targeted strategies in PD, AD, and MS. In PD, probiotics, prebiotics, symbiotics, postbiotics, and fecal microbiota transplantation can restore dysbiotic microbial communities, increase SCFA production, and support dopaminergic neuron survival via GBA-mediated mechanisms (Cryan et al., 2012; Westfall et al., 2017). Butyrate, for instance, increases glucocorticoid receptor expression in the gut, modulates inflammation, and exerts neuroprotective effects (Fukumoto et al., 2003; Gareau et al., 2008; Clarke et al., 2012). These interventions can attenuate neuroinflammatory pathways, including Toll-like receptor 4/nuclear factor kappa B and mitogen-activated protein kinase signaling, with preclinical studies showing improvements in gastrointestinal and neuronal health, although large-scale clinical trials are limited (Ma et al., 2024).

In AD, SCFAs produced by gut microbiota through dietary fiber fermentation influence neuroinflammation and amyloid-beta and tau pathology, providing mechanistic links between microbial metabolites and neurodegeneration (Brinton 2009; Harach et al., 2017; Wu et al., 2021; Lei et al., 2025). Mechanistically, SCFAs can cross the blood–brain barrier and influence microglial maturation and function, as shown by normalization of microglial homeostasis in germ-free mice upon SCFA supplementation (Erny et al., 2015). Additional studies indicate that butyrate suppresses LPS-induced nuclear factor kappa B activation in microglia through histone deacetylase inhibition, thereby attenuating neuroinflammation (Huuskonen et al., 2004; Caetano-Silva et al., 2023).

Similarly, in MS, microbiome-targeted interventions enhance regulatory T cell activity, suppress Th1/Th17 responses, and reduce autoimmune-mediated neuroinflammation, complementing hormone-based approaches to promote remyelination and neuroprotection (Berer et al., 2017; Mirza et al., 2017; Tsogka et al., 2023).

Hormone-targeted strategies can further modulate the GBA and neuronal outcomes. Estrogens improve gut barrier integrity, reduce bacterial translocation, and regulate tight junction proteins, indirectly mitigating neuroinflammation and supporting cognitive function (Brinton, 2009; Braniste et al., 2014; Chelakkot et al., 2018). Progesterone promotes Lactobacillus growth, reduces gut permeability, suppresses pro-inflammatory cytokines, and exerts neuroprotective effects via PGRMC1 receptor activation (Coronel et al., 2016; Zhou et al., 2019; Stegemann et al., 2023). Glucocorticoids may increase gut permeability and inflammation, potentially exacerbating neurodegenerative processes (Fasano, 2011; Tena-Garitaonaindia et al., 2022). Testosterone modulates microbial diversity, SCFA production, immune tolerance, and gut microbial composition, indirectly affecting neuronal function (Canfora et al., 2015; Ben-Batalla et al., 2020; Campos-Perez et al., 2021; Song et al., 2023). Clinical studies indicate that hormone replacement therapy, for example in premature ovarian insufficiency, can normalize gut microbiome and serum metabolome alterations, highlighting avenues to modulate neurodegenerative risk via microbiome-hormone interactions (Westfall et al., 2017; Jiang et al., 2021). Beyond systemic effects, estrogens exert direct neuroprotective actions in the brain. 17β-estradiol promotes neuronal survival and synaptic plasticity by upregulating brain-derived neurotrophic factor expression via estrogen response elements in the brain-derived neurotrophic factor gene promoter (Sohrabji et al., 1995), and by activating PI3K/Akt and mitogen-activated protein kinase/ERK signaling cascades through both genomic and membrane-associated estrogen receptors (Watters et al., 1997).

Collectively, these findings provide a strong rationale for integrating microbiome modulation with hormone-targeted strategies to develop disease-specific interventions. While much evidence remains preclinical, the converging mechanisms between the GBA, sex hormones, and neurodegeneration underline the potential of these approaches to improve outcomes in PD, AD, and MS, warranting further translational studies in humans (Brinton, 2009; Cryan et al., 2012; Westfall et al., 2017).

## Conclusion

**Figure 4:**
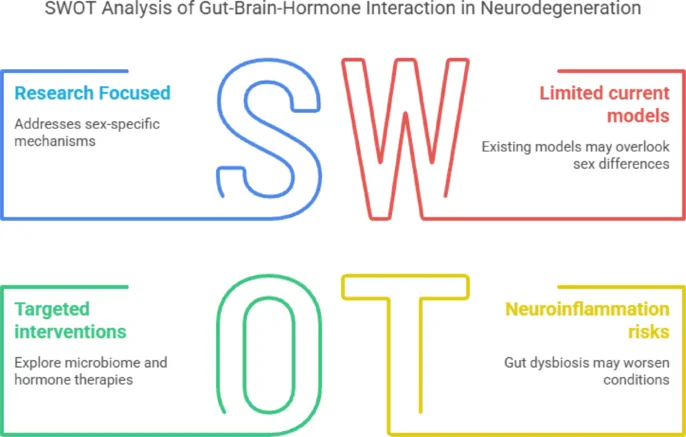
SWOT analysis. SWOT analysis summarizing Strengths (S, internal positive factors), Weaknesses (W, internal negative factors), Opportunities (O, external positive factors), and Threats (T, external negative factors) related to gut–brain–hormone interactions in neurodegeneration. Created with Napkin.AI.

The gut-brain-hormone interaction plays a pivotal role in the development and progression of neurodegenerative diseases. Gut dysbiosis and hormonal decline contribute to neuroinflammation, driving neuronal damage and disease progression. Sex-specific pathways, influenced by variations in hormone levels and immune responses, result in differences between men and women in neurodegenerative diseases. Addressing the sex bias in research, adapting disease models for sex-specific mechanisms, and exploring microbiome-based and hormone-targeted interventions are critical steps toward developing effective therapeutic strategies. By integrating hormonal and sex-specific aspects of the GBA, we can advance our understanding of neurodegenerative diseases and improve health outcomes for all sexes (**Figure 4**). The interplay between steroid hormones, the gut microbiome, and the GBA offers promising avenues for novel therapeutic strategies in neurodegenerative diseases. This emerging cross-disciplinary field has gained significant momentum since 2020 and new developments, such as single-cell mapping of sex-hormone receptors, e.g., in the vagus nerve, may further elucidate these mechanisms and require future updates to this field (Mendez‐Hernandez et al., 2025).

## Data Availability

*Not applicable.*
